# Menin and p53 have non-synergistic effects on tumorigenesis in mice

**DOI:** 10.1186/1471-2407-12-252

**Published:** 2012-06-18

**Authors:** Kelly A Loffler, Arne W Mould, Paul M Waring, Nicholas K Hayward, Graham F Kay

**Affiliations:** 1 Queensland Institute of Medical Research, 300 Herston Road, Herston, QLD, 4006, Australia; 2 Department of Pathology, University of Melbourne, Parkville, VIC, 3010, Australia; 3 Present address: William Dunn School of Pathology, University of Oxford, Oxford, UK

## Abstract

**Background:**

While it is now more than a decade since the first description of the gene mutation underlying the tumour predisposition syndrome multiple endocrine neoplasia type 1 (MEN1), the mechanism by which its protein product menin acts to prevent development of tumours is still poorly understood.

**Methods:**

We undertook a genetic experiment to assess whether menin synergises with p53. Mice carrying various combinations of *Men1* and *Trp53* mutations were generated then survival and pathology assessed.

**Results:**

While homozygous loss of *Trp53* in mice resulted in early onset, aggressive tumours and profoundly reduced lifespan, heterozygous loss of either *Trp53* or *Men1* caused later onset disease, with a spectrum of tumours characteristic of each tumour suppressor gene. Loss of one copy of *Men1* in animals also lacking both alleles of *Trp53* did not exacerbate phenotype, based on survival, animal weight or sites of pathology, compared to *Trp53* deletion alone. Dual heterozygous deletion of *Men1* and *Trp53* resulted in a small reduction in lifespan compared to the individual mutations, without new tumour sites. In the adrenal, we observed development of cortical tumours in dual heterozygous animals, as we have previously seen in *Men1*^+/−^ animals, and there was loss of heterozygosity at the *Men1* allele in these tumours. Median number of pathology observations per animal was increased in dual heterozygous animals compared with heterozygous loss of *Trp53* alone.

**Conclusions:**

Simultaneous heterozygous deletion of *Men1* in animals with either heterozygous or homozygous deletion of *Trp53* did not result in formation of tumours at any new sites, implying additive rather than synergistic effects of these pathways. Mice that were *Men1*^+/−^ in addition to *Trp53*^+/−^ had tumours in endocrine as well as other sites, implying that increase in total tumour burden, at sites typically associated with either *Men1* or *Trp53* loss, contributed to the slight decrease in survival in *Men1*^+/−^: *Trp53*^+/−^ animals in comparison with their littermates.

## Background

Menin, the tumour suppressor product of the *MEN1* gene that is mutated in the human tumour predisposition syndrome multiple endocrine neoplasia type 1 (MEN1), is a 610 amino acid protein with no homology to any known proteins [1,2]. Menin localises predominantly to the nucleus, and is associated with a variety of proteins that implicate it in signal transduction pathways, as a transcriptional co-regulator, and in regulation of chromatin [3-5]. One of the proteins that associates with menin is the Fanconi anaemia protein FANCD2 [6], which is involved in response to DNA damage and participates in the BRCA/p53 pathway of tumour suppression [7].

Both spontaneous and familial cases of human *MEN1* mutation result in predisposition to a range of tumour types, predominantly in endocrine organs such as parathyroid, pituitary and enteropancreatic sites [8]. Several groups have published mouse models of MEN1, which replicate the human syndrome and result in tumour development, most commonly in endocrine pancreas, pituitary, parathyroid and thyroid glands, adrenals, in the gonads in some models, as well as other rarer sites [9-12].

Mutation of the *TRP53* gene encoding p53 has been associated with many tumour types [for recent review of p53 biology see [13]. Sporadic somatic mutations are commonly detected in different cancer types, and occur along the length of the gene. Hereditary *TRP53* mutations are associated with Li Fraumeni syndrome (LFS), characterized by autosomal dominant inheritance and early onset tumour development, multiple tumours within an individual, and multiple affected family members. The most frequent tumour types in LFS are soft tissue sarcomas and osteosarcomas, breast cancer, brain tumours, leukaemia, and adrenocortical carcinoma (OMIM 151623). Mouse models of *Trp53* deletion also exhibit a range of cancer types, particularly sarcoma and lymphoma [14,15].

Our previous experiments have indicated that compound heterozygous loss of both *Men1* and *Rb1* genes has little effect on the rate, severity or onset of tumorigenesis, or on the spectrum of observed tumour types compared to individual gene deletions [16]. In contrast, the comparable cross of animals with heterozygous loss of both *Rb1* and *Trp53* genes resulted in a marked increase in severity, decreased median lifespan, and the occurrence of novel tumour types not observed in either of the individual knockout animals [17,18]. *Trp53* can act synergistically with *Brca2*[19] and *Cdkn2c*[20], a known menin-regulated gene [5,21]. p18, the protein product of *Cdkn2c*, also collaborates with menin to suppress neuroendocrine tumorigenesis [22]. We therefore sought to ascertain whether a similar genetic interaction would be observed between *Men1* and *Trp53*.

## Methods

Mice with a targeted disruption of exon 2 of *Men1* have been previously described, and were genotyped by PCR from tail or ear biopsy DNA [11]. Mice with targeted deletion of *Trp53* obtained from Tyler Jacks were maintained and genotyped as previously described [15]. A cohort of animals including various combinations of the two tumour suppressor gene deletions were generated by cross-breeding and maintained under authorisation from the Queensland Institute of Medical Research Animal Ethics Committee in compliance with the Australian Code of Practice for the Care and Use of Animals for Scientific Purposes, in standard housing conditions with 12 hour light–dark cycle with food and water available *ad libitum*. Animals of the expected range of genotypes were born and viable, and all initially appeared normal and healthy. Cohorts were subsequently monitored periodically for signs of illness or overt tumour development. Upon observation of morbidity, animals were euthanased by CO_2_ asphyxiation, weighed, and a full necropsy carried out. At 21 months the experiment was terminated and all remaining animals culled and necropsies performed. At necropsy any pathology such as tumours or other grossly visible abnormalities were noted. Tissues were rinsed in PBS then fixed overnight in 10% neutral buffered formalin, washed in 70% ethanol and re-assessed using a stereomicroscope to confirm observations. Age was calculated using the date of birth and date of death to calculate the age in days, then dividing this value by 30.416667 to approximate the age in months. Survival was plotted in GraphPad Prism 5.01 for Windows, and Kaplan-Meier survival curves were analysed using Gehan-Breslow-Wilcoxon tests (GraphPad Software, San Diego U.S.A.). P values less than 0.05 were considered significant. Whole animal weights in grams were compared by one way ANOVA with Tukey’s correction for multiple comparisons. Pathology data from necropsy observations was tabulated by genotype group then analysed using PASW Statistics 17.0 (SPSS Inc., Chicago U.S.A.). Loss of heterozygosity was assessed by isolation of genomic DNA from formalin fixed tissues, followed by genotyping PCR as previously described [16]. Products were separated by agarose gel electrophoresis, photographed and band intensities calculated using ImageJ image analysis software (NIH). Paraffin embedding and hematoxylin and eosin staining of tissues was carried out by the University of Queensland-Queensland Institute of Medical Research Histotechnology Facility, and sections were scanned using an Aperio Scanscope XT (Aperio Technologies, Vista U.S.A.). Histopathological assessment of adrenal sections was carried out a histopathologist (PMW), who was blinded to the genotypes of the animals.

## Results

### Survival analyses

**Figure 1:**
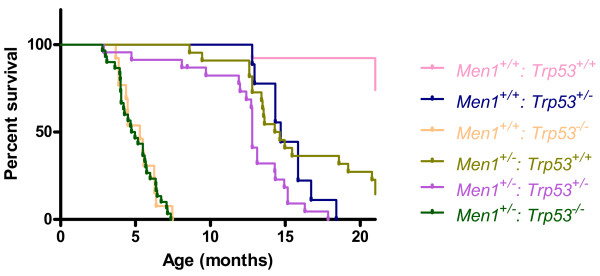
**Survival analysis of animals by genotype.** Animals were culled on noting morbidity or distress, or at 21 months of age, and full necropsies carried out. Presence of any kind of overt gross pathology, including tumours or other abnormalities, was scored as an event. Data is plotted as a cumulative surviving percentage of the cohort of animals, versus age in months.

Survival analysis indicated that homozygous deletion of *Trp53* resulted in dramatically shortened lifespan compared to wild-type mice, however the further loss of one allele of *Men1* had no significant modifying effect on median survival (*Men1*^+/+^: *Trp53*^−/−^ median 5.29 months, mean 5.192 months, SD 1.172; *Men1*^+/−^: *Trp53*^−/−^ median 4.85 months, mean 5.016 months, SD 1.287) (Figure 1). Mice with heterozygous deletion of either *Men1* or *Trp53* had decreased survival compared to their wild-type littermates. Combined heterozygous deletion of both *Men1* and *Trp53* resulted in further reduction in median lifespan of almost two months compared to either of the individual mutants (*Men1*^+/−^: *Trp53*^+/+^ median 14.47 months, mean 15.76 months, SD 4.061; *Men1*^+/+^: *Trp53*^+/−^ median 14.70 months, mean 15.10 months, SD 1.804; *Men1*^+/−^: *Trp53*^+/−^ median 12.79 months, mean 12.22 months, SD 3.479; p < 0.02). Most of the *Men1*^+/+^: *Trp53*^+/+^ animals survived in apparent health until 21 months old, at which time all remaining animals were culled and the experiment ended (median age 21.14 months, mean 19.10 months, SD 3.756).

### Gross pathology observations

As was expected based on our previous analyses of mice lacking one copy of *Men1*, the main pathologies observed in the *Men1*^+/−^: *Trp53*^+/+^ cohort were in the pancreas, pituitary, gonads, and adrenal glands (Table 1). Gross pathologies observed in our cohort of *Men1*^+/+^: *Trp53*^+/−^ and *Men1*^+/+^: *Trp53*^−/−^ animals were consistent with published descriptions of other *Trp53* knockouts, most commonly including lymph node and spleen pathologies, consistent with the frequent lymphoma observed previously by others. *Men1*^+/−^: *Trp53*^−/−^ animals showed essentially the same range of gross pathology that was observed in *Men1*^+/+^: *Trp53*^−/−^ animals. *Men1*^+/−^: *Trp53*^+/−^ animals presented with a range of gross pathologies consistent with the phenotypes observed in the individual mutants. No tumours were observed in organs that were not affected in either of the single mutants. Comparisons for each organ or site of pathology between genotype groups (Table 1) indicated that there were no statistically significant differences between *Men1*^+/−^: *Trp53*^−/−^ and *Men1*^+/+^: *Trp53*^−/−^ animals in tumorigenesis rates at any site. There were also no significant differences between *Men1*^+/−^: *Trp53*^+/+^ and *Men1*^+/−^: *Trp53*^+/−^ cohorts at any site. In a few organs there were however differences between *Men1*^+/+^: *Trp53*^+/−^ and *Men1*^+/−^: *Trp53*^+/−^ cohorts (Two sided Fisher’s Exact Tests: pituitary p = 0.011, pancreas p = 0.045, uterus p = 0.038, other sites not significant).

**Table 1:** Pathology observed grossly, by anatomical site

	***Men1***^**+/+**^**;*Trp53***^**+/+**^		***Men1***^**+/+**^**;*Trp53***^**+/−**^		***Men1***^**+/+**^**;*Trp53***^**−/−**^		***Men1***^**+/−**^**;*Trp53***^**+/+**^		***Men1***^**+/−**^**;*Trp53***^**+/−**^		***Men1***^**+/−**^**;*Trp53***^**−/−**^	
*Pituitary*	4/13	(30.8%)	0/9	0	1/13	(7.7%)	14/19	(73.7%)	11/21	(52.4%)	5/28	(17.9%)
*Pancreas*	0/13	0	1/9	(11.1%)	1/13	(7.7%)	13/19	(68.4%)	12/22	(54.5%)	4/28	(14.3%)
*Thyroid*	3/11	(27.3%)	0/8	0	0/11	0	5/17	(29.4%)	9/22	(40.9%)	4/27	(14.8%)
*Parathyroid*	1/11	(9.1%)	1/8	(12.5%)	0/11	0	4/17	(23.5%)	5/22	(22.7%)	0/27	0
*Thymus*	0/13	0	0/9	0	3/13	(23.1%)	1/19	(5.9%)	2/22	(9.1%)	11/28	(39.3%)
*Lymph Nodes*	2/13	(15.3%)	3/9	(33.3%)	9/13	(69.2%)	4/19	(21.1%)	9/22	(40.1%)	20/28	(71.4%)
*Spleen*	1/13	(7.7%)	1/9	(11.1%)	7/13	(53.8%)	3/19	(15.8%)	4/22	(18.2%)	21/28	(75.0%)
*Muscle*	0/13	0	1/9	(11.1%)	5/13	(38.5%)	1/19	(5.3%)	3/22	(13.6%)	6/28	(21.4%)
*Bone*	0/13	0	3/9	(33.3%)	0/13	0	3/19	(15.8%)	2/22	(9.1%)	1/28	(3.6%)
*Bladder/Urethra*	0/13	0	2/9	(22.2%)	0/13	0	0/19	0	0/22	0	1/27	(3.7%)
*Prostate/Seminal Vesicles*	2/5	(40.0%)	2/6	(33.3%)	1/5	(20.0%)	1/9	(11.1%)	4/12	(33.3%)	1/15	(6.7%)
*Testes*	0/5	0	1/6	(16.7%)	1/5	(20.0%)	5/9	(55.6%)	7/12	(58.3%)	2/16	(12.5%)
*Uterus*	3/8	(37.5%)	2/3	(66.7%)	0/8	0	2/10	(20.0%)	0/10	0	0/11	0
*Ovaries*	0/8	0	1/3	(33.3%)	1/8	(12.5%)	5/10	(50.0%)	6/10	(60.0%)	5/11	(45.5%)
*Mammary Glands*	1P/13	(7.7%)	0/9	0	0/6	0	1 L/18	(5.6%)	1 L,1P/17 (5.9%L,5.9%P)		0/14	0
*Adrenal Glands*	0/13	0	1/8	(12.5%)	3/13	(23.1%)	5/18	(27.8%)	9/21	(42.6%)	6/28	(21.4%)
*Lung*	3/13	(23.1%)	1/9	(11.1%)	3/13	(23.1%)	2/19	(10.5%)	4/21	(19.0%)	3/28	(10.7%)
*Liver*	1/13	(7.7%)	3/9	(33.3%)	3/13	(23.1%)	1/19	(5.3%)	2/21	(9.5%)	6/28	(21.4%)

Data are shown as "Number of animals with abnormal or pathology observation"/"Total number of animals" (percentage of cohort), tabulated by genotype group. For mammary observations, L indicates appearance similar to lactation as seen during normal pregnancy or suckling, as we have previously observed in some animals with pituitary prolactinoma, P indicates other pathology.

### Pancreatic pathology observations

**Figure 2:**
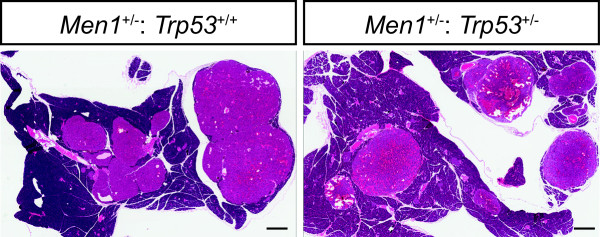
**Pancreatic pathology.** Haematoxylin and eosin stained sections from the pancreata of littermate *Men1*^+/−^: *Trp53*^+/+^ and *Men1*^+/−^: *Trp53*^+/−^ animals as indicated (both between 12 and 13 months old at time of necropsy). Bars represent 500 μm.

As has been previously observed, pancreatic endocrine pathology is frequently observed in *Men1*^+/−^ animals [9-12], and in our *Men1*^+/−^ animals was histologically described as adenoma, which were typically immunopositive for insulin [11]. Gross pathology was observed at similar rates in the *Men1*^+/−^: *Trp53*^+/+^ and *Men1*^+/−^: *Trp53*^+/−^ cohorts here, as discussed above. Histologically, the appearance of islet tumours in *Men1*^+/−^: *Trp53*^+/−^ animals was not distinguishable from that we have observed in the *Men1*^+/−^: *Trp53*^+/+^ animals at a comparable age. For example, Figure 2 shows haematoxylin and eosin stained sections from the pancreas of *Men1*^+/−^: *Trp53*^+/+^ and littermate *Men1*^+/−^: *Trp53*^+/−^ animals, including some large, highly vascular adenomas, but notably some smaller and histologically normal islets are still apparent in each of these animals. Consistent with the younger age at necropsy in *Men1*^+/−^: *Trp53*^−/−^ animals, islets in these animals were sometimes hyperplastic but had rarely progressed to frank adenoma (not shown).

### Adrenal pathology observations

**Figure 3:**
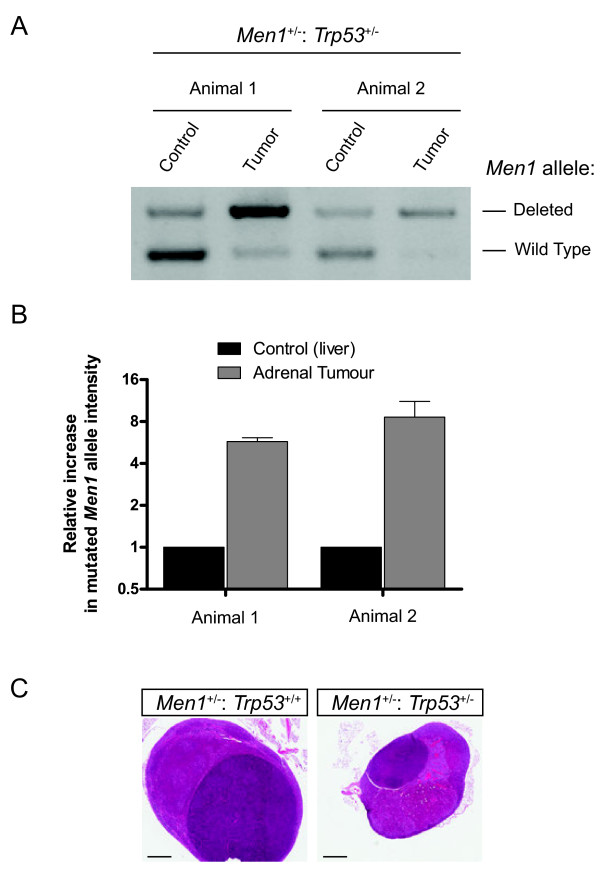
**Adrenal pathology.** PCR was used to assess the representation of wild type and mutated alleles of *Men1* in genomic DNA from adrenal tumours, and from liver from the same animal as a control (**A**). Results of these assays were quantified and the relative representation of the mutated compared to the wild type allele in tumours was normalised to the band intensities in the liver of the same animal. Mean (+SE) of triplicate experiments is shown in (**B**). (**C**) Histopathology of adrenal glands from litter mate *Men1*^+/−^: *Trp53*^+/+^ and *Men1*^+/−^: *Trp53*^+/−^ animals (14 and 12 months old respectively at necropsy) as indicated, showing similar cortical hyperplasia and adenoma development. Bars represent 500 μm.

Adrenal pathology is sometimes observed in *Men1*^+/−^ animals [9-12], and was apparent at a statistically similar rate in our *Men1*^+/−^: *Trp53*^+/−^ cohort. We obtained genomic DNA from some of these adrenal tumours, and by comparison with normal tissue (from the liver of the same animal), observed that these adrenal tumours had undergone loss of heterozygosity at the *Men1* locus, with relative over-representation of the deleted allele (Figure 3 A and B).

Histological analysis revealed that large adrenal tumours in several *Men1*^+/−^: *Trp53*^+/−^ animals were cortical adenomas or carcinomas, similar to those we had previously observed in our analysis of *Men1*^+/−^ animals [11]. Figure 3C shows adrenal histology from litter mate *Men1*^+/−^: *Trp53*^+/+^ and *Men1*^+/−^: *Trp53*^+/−^ animals (14 and 12 months old respectively), in which both animals developed similar cortical adenoma and hyperplasia. In two *Men1*^+/−^: *Trp53*^−/−^ animals in which pathology was grossly observed in the adrenal, histological assessment showed one of these to be a teratocarcinoma, and another was a poorly differentiated malignant neoplasm of uncertain origin, most likely an undifferentiated cortical carcinoma.

### Animal weight

**Figure 4:**
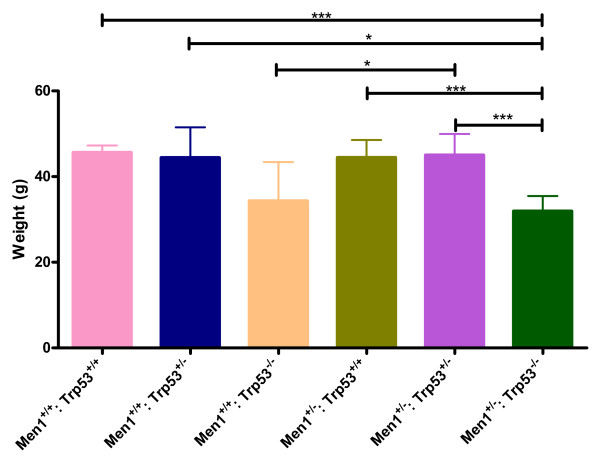
**Animal weight at necropsy by genotype.** Mean whole animal weight at necropsy (grams), by genotype group. Error bars represent SE. * p < 0.05, ***p < 0.001 by ANOVA with Tukey’s test.

We also compared weight between genotype groups (Figure 4). The *Men1*^+/+^: *Trp53*^−/−^ (mean 34.36 g, range 21.7 – 52.5) and *Men1*^+/−^: *Trp53*^−/−^ (mean 31.98 g, range 15.1 – 44.4) animals, while not significantly different from each other, were generally lighter than the other genotype cohorts. There were no significant differences in weight between *Men1*^+/−^: *Trp53*^+/+^ (mean 44.49 g, range 25.9 – 59.0), *Men1*^+/+^: *Trp53*^+/−^ (mean 44.44 g, range 28.3 – 54.1), *Men1*^+/−^: *Trp53*^+/−^ (mean 45.07 g, range 27.0 – 61.5) and *Men1*^+/+^: *Trp53*^+/+^ (mean 45.68 g, range 37.3 – 53.4) cohorts, indicating that general growth and development of these animals was not grossly disrupted.

### Occurrence of multiple pathology observations

**Figure 5:**
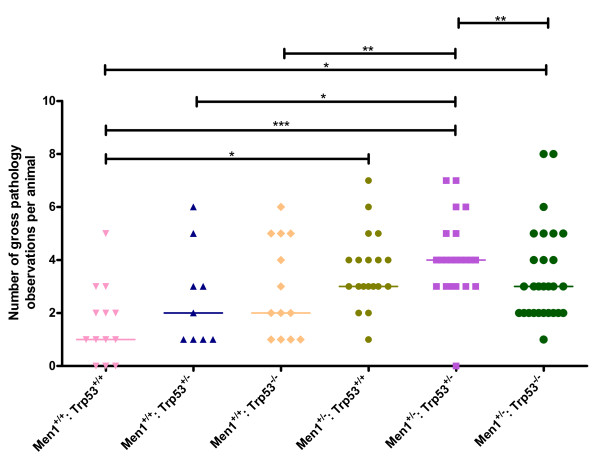
**Number of pathology observations per animal by genotype.** Data from observations at necropsy were recorded and the total number of pathological/abnormal observations per animal compiled. Data are plotted by genotype as indicated, with each individual animal represented as a dot point, and median number of observations per animal for each genotype group indicated by a horizontal line. * p < 0.05, **p < 0.01, ***p < 0.001, Fisher’s exact test.

A defining feature of both MEN1 and Li Fraumeni syndromes is the occurrence of multiple tumours, and a possible explanation for the decreased lifespan of the dual heterozygous animals compared to either of the individual lines may be increased overall tumour burden. This could be indicated by a higher total number of pathology observations per animal. As shown in Figure 5, pathology observations were recorded in multiple organs for most animals in all cohorts, including some of the wild-type littermates (*Men1*^+/+^: *Trp53*^+/+^), which generally reached very advanced age but nevertheless had often developed several minor pathologies. Comparison of the number of scored “pathological” observations per animal indicated that *Men1*^+/−^: *Trp53*^+/−^ animals presented with a median of four pathological events compared to two for *Men1*^+/+^: *Trp53*^+/−^ animals (p = 0.016, Fisher’s Exact Test) or three in the *Men1*^+/−^: *Trp53*^+/+^ cohort (p = 0.715, Fisher’s Exact Test). *Men1*^+/−^: *Trp53*^−/−^ animals showed a median of three observed pathologies while *Men1*^+/+^: *Trp53*^−/−^ animals showed two (p = 0.183, Fisher’s Exact Test).

## Discussion

Homozygous deletion of the mouse *Men1* gene is lethal during embryogenesis [9,23], while homozygous deletion of *Trp53* in mice results in rapid onset development of multiple tumours and severely shortened lifespan compared to wild-type animals, or even to animals with heterozygous deletion of *Trp53*[14,15]. Compound heterozygous *Men1*^+/−^: *Trp53*^+/−^ animals described here were viable into adulthood, and median survival was much longer than the corresponding cohort of animals with homozygous deletion of *Trp53*, indicating that losing one copy each of both *Men1* and *Trp53* is not biologically equivalent to losing both copies of *Trp53* or of *Men1*.

Animals with homozygous deletion of *Trp53*, particularly those which also had deletion of one copy of *Men1*, had lower whole body weights at necropsy, which we attribute primarily to the much younger age at which these animals showed onset of disease symptoms. A secondary contributing factor may be that when the *Men1*^+/+^: *Trp53*^−/−^ and *Men1*^+/−^: *Trp53*^−/−^ animals became ill they appeared to lose condition rapidly, and thus by the time of necropsy may have lost weight compared to remaining animals that were still healthy at the same age. This may be comparable to the wasting or cachexia often observed in human cancer patients.

We monitored the occurrence of tumours in these cohorts of animals and found that an endocrine pattern of tumorigenesis similar to that seen in MEN1 syndrome in humans, and in mice with heterozygous deletion of *Men1*, is still apparent when combined with heterozygous loss of p53. The tumour types associated with LFS in humans with heterozygous loss of *TRP53* are not perfectly recapitulated by heterozygous or homozygous loss of *Trp53* in mice, however each of these is associated with its own particular spectrum of tumour development [14,15]. We have noted that each of these spectra is also retained in combination with *Men1*^+/−^. We thus conclude that mutations affecting either p53 or menin are associated with particular cellular consequences which correspond to propensity for tumorigenesis in particular tissues, and that additional mutations affecting the other pathway have negligible effect in terms of tissue specificity of tumour development. The absence of additional tumour sites is in contrast to previous descriptions of genetic interaction between *Trp53* and *Rb1* in which tumours develop in additional sites as well as with increased severity, resulting in dramatically shortened lifespan [17,18].

Notably, the sites where pathology frequencies varied between *Men1*^+/+^: *Trp53*^+/−^ and *Men1*^+/−^: *Trp53*^+/−^ cohorts included the pancreas and pituitary, which are typical sites of *Men1*-associated tumorigenesis in mice, and the uterus, which may be affected by alterations in hormones secondary to pituitary pathology.

## Conclusions

Both Li-Fraumeni and MEN1 syndromes are associated with high risk of development of multiple tumours (OMIM 151623 and 131100). This is also the case in both *Men1*^+/−^ and *Trp53*^+/−^ mice, as we and others have observed previously and confirmed here. The observation of highest median number of pathology observations per animal in the *Men1*^+/−^: *Trp53*^+/−^ cohort indicates that tumours at more sites, likely corresponding to increased total tumour burden, was the cause underlying the steeper slope of the survival curve for these animals in comparison with either mutation alone. This resulted in the shortening of median lifespan for the *Men1*^+/−^: *Trp53*^+/−^ cohort.

Mice with a combination of mutations affecting p53 and menin retain both of the tissue specific patterns of tumorigenesis associated with each of these tumour suppressors. They develop cancers in the endocrine pattern typically associated with MEN1 [8-12] and the sarcoma/lymphoma constellation that has been typically linked to the p53 pathway [14,15]. These data imply that menin and p53 have independent and non-synergistic effects on tumorigenesis.

We cannot exclude the possibility that these mutations have consequences that result in changes in biology at a molecular level in individual cells, however this is not apparent at the level of gross tumour development. This implies some level of plasticity or compensation, or of independent pathways of activity for menin and p53.

We postulate that the increased total tumour burden, without new tumour types, in the compound *Men1*^+/−^: *Trp53*^+/−^ cohort underlies the slightly shorter median survival of the compound heterozygous animals, compared to heterozygous loss of either of these tumour suppressor genes alone.

*TRP53* is very frequently mutated in many types of tumours, however comprehensive sequencing of exomes of sporadic human pancreatic neurendocrine tumours identified mutations in *Trp53* in only two of 68 tumours [24]. The molecular pathway by which p53 acts to prevent tumour development can be disrupted in cancers by multiple mechanisms, not only by sequence mutation of the gene itself [for example [25] but the experiment described here provides genetic confirmation that disruption of the broader p53 pathway is unlikely to be important in these endocrine tumour types. Therapeutic avenues targeting the p53 pathway are therefore likely to be of little clinical utility in treatment of such tumours.

## Competing interests

The authors declare that they have no competing interests.

## Authors' contributions

AWM, NKH and GFK designed and planned the experiments. AWM and KAL carried out all animal work. KAL carried out LOH analyses, compiled data and performed statistical analyses and wrote the manuscript. PMW assessed adrenal histopathology. All authors assisted with drafting the manuscript, and read and approved the final manuscript.

## Pre-publication history

The pre-publication history for this paper can be accessed here:

http://www.biomedcentral.com/1471-2407/12/252/prepub
